# Targeting Reprogrammed Cancer-Associated Fibroblasts with Engineered Mesenchymal Stem Cell Extracellular Vesicles for Pancreatic Cancer Treatment

**DOI:** 10.34133/bmr.0050

**Published:** 2024-08-02

**Authors:** Pengcheng Zhou, Xian’guang Ding, Xuanlong Du, Lianhui Wang, Yewei Zhang

**Affiliations:** ^1^ School of Medicine, Southeast University, Nanjing 210000, China.; ^2^ Department of General Surgery, Affiliated Hospital of Nantong University, Nantong 226001, China.; ^3^ State Key Laboratory of Organic Electronics and Information Displays & Jiangsu Key Laboratory for Biosensors, Institute of Advanced Materials (IAM), Nanjing University of Posts and Telecommunications, Nanjing 210023, China.; ^4^ Hepatobiliary and Pancreatic Center, The Second Affiliated Hospital of Nanjing Medical University, Nanjing 210011, China.

## Abstract

**Background:** As one of the most aggressive and lethal cancers, pancreatic cancer is highly associated with cancer-associated fibroblasts (CAFs) that influence the development and progression of cancer. Targeted reprogramming of CAFs may be a promising strategy for pancreatic cancer. This study aims to construct engineered extracellular vesicles (EVs) with surface modification of integrin α5 (ITGA5)-targeting peptide and high internal expression of miR-148a-3p by endogenous modification for targeted reprogramming of pancreatic CAFs. **Methods:** Bone marrow mesenchymal stem cells (BMSCs) and pancreatic CAFs were cocultured to examine the effect of BMSC-derived EVs on the expression levels of CAF markers. miR-148a-3p was identified as a functional molecule. The mechanism of miR-148a-3p was elucidated using the dual-luciferase reporter assay. BMSCs were infected with TERT-encoding and miR-148a-3p-encoding lentiviruses. Subsequently, BMSCs were modified with ITGA5-specific targeting peptide. The supernatant was ultracentrifuged to obtain the engineered EVs (ITGA5-EVs^-148a^), which were used to reprogram CAFs. **Results:** BMSCs modulated CAF marker expressions through EVs. miR-148a-3p was up-regulated in BMSCs. The expression of miR-148a-3p in pancreatic CAFs was down-regulated when compared with that in normal fibroblasts (NFs). Mechanistically, ITGA5-EVs^-148a^ effectively suppressed the proliferation and migration of pancreatic CAFs by targeting ITGA5 through the TGF-β/SMAD pathway. ITGA5-EVs^-148a^ was associated with enhanced cellular uptake and exhibited enhanced in vitro and in vivo targeting ability. Moreover, ITGA5-EVs^-148a^ exerted strong reconfiguration effects in inactivating CAFs and reversing tumor-promoting effects in 3D heterospheroid and xenograft pancreatic cancer models. **Conclusions:** This targeted CAF reprogramming strategy with genetically engineered ITGA5-EVs^-148a^ holds great promise as a precision therapeutics in clinical settings.

## Introduction

Pancreatic cancer is the deadliest cancer with a 5-year survival rate of less than 10% [1,2]. Most patients with pancreatic cancer are diagnosed with advanced-stage cancer as they do not exhibit early symptoms and are not eligible for surgical treatment. Standard chemotherapy regimens for PDAC (pancreatic ductal adenocarcinoma) patients such as nab-paclitaxel and gemcitabine and FOLFIRINOX offer only a month-long survival benefit and usually display chemoresistance and side effects [3]. Therefore, it is urgent to develop effective therapeutic strategies for pancreatic cancer.

A typical histopathological feature of pancreatic cancer is the dense fibrotic stroma, which accounts for more than 80% of the tumor mass [4]. The increased proportion of stroma in patients with pancreatic cancer predicts poor survival outcomes [5]. The stroma of pancreatic cancer mainly comprises extracellular matrix (ECM) and cancer-associated fibroblasts (CAFs). Activated pancreatic stellate cells (PSCs) are the major sources of CAFs and produce large amounts of ECM [6]. The desmoplastic stroma in a tumor niche increases the pressure of the fluid between cells, compressing blood vessels, preventing the delivery of therapeutics to the target area through circulation, and consequently reducing therapeutic effectiveness. In addition to remodeling the stroma, CAFs secrete various paracrine factors that promote cancer cell proliferation, migration, invasion, metastasis, and chemoresistance. Thus, to develop novel therapeutic strategies for pancreatic cancer, it is crucial to consider the effect of CAFs on the pancreatic microenvironment.

Bone marrow mesenchymal stem cells (BMSCs) are potential candidates in treating pancreatic cancer. Recent advances in pancreatic cancer research have identified the relationship between BMSCs and pancreatic cancer and established the “bone marrow–pancreatic cancer axis” theory [7]. BMSCs can exert growth-inhibitory effects against pancreatic cancer [8] and hepatic cancer [9]. Additionally, external BMSC administration exerts effective therapeutic effects on fibrotic diseases [10,11]. However, the safety and efficiency of mesenchymal stem cells (MSCs) is controversial. For example, homologous MSC production is time consuming, costly, and associated with storage challenges and senescence during cell expansion. In comparison, BMSC-derived extracellular vesicles (EVs) not only inherit similar cellular capacity but also feature lower oncogenicity, excellent biocompatibility, and long-circulating ability [12]. BMSC-derived EVs suppress the progression of various cancers [13,14]. Preclinical studies have demonstrated that BMSC-derived EVs can exert antifibrotic effects through multiple mechanisms [15–17]. Despite these promises, current EV therapeutics are severely curtailed by their poor targeting capacity and compromised therapeutic efficacy.

MicroRNAs (miRNAs), which are a class of endogenous small noncoding RNAs, negatively regulate gene expression by inducing translational repression or mRNA decay [18]. In pancreatic cancer, miRNA dysregulation is associated with a broad spectrum of cellular processes. Additionally, miRNAs are reported to regulate the phenotype of CAFs [19,20]. miR-148a functions as an antioncogene in various human tumors [21,22]. The miR-148a and miR-148b levels are down-regulated in CAFs of endometrial cancer. The loss of miR-148a or miR-148b in CAFs is reported to promote the invasion and metastasis of endometrial cancer cells [23,24]. miR-148a, which is enriched in MSC-derived EVs, can target KLF6 to reprogram the proinflammatory phenotype of macrophages to the anti-inflammatory phenotype by inhibiting the signal transducer and activator of transcription 3 (STAT3) pathway and consequently can alleviate liver fibrosis [25]. These findings indicate that miR-148a is a novel candidate for gene therapy to modulate CAFs for pancreatic cancer treatment.

**Fig. 1.**
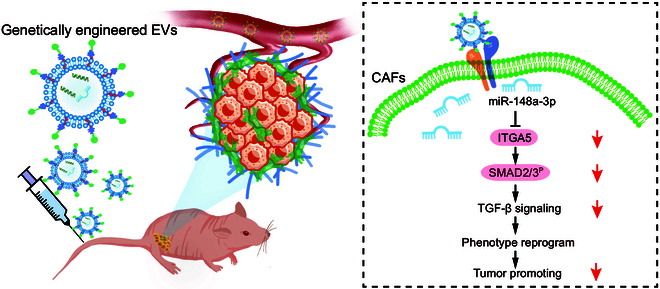
The schematic diagram illustrates the process of EVs targeting CAFs and reprogramming CAFs.

In this study, we clinically revealed that pancreatic CAFs feature a very low expression of miR-148a-3p. Next, the functional roles of miR-148a-3p in CAFs were examined and a reliable target that can be used for targeting CAFs was identified. BMSCs were engineered with miR-148a-3p-encoding lentivirus, and the membrane was incorporated with ITGA5-targeting peptides to obtain genetically engineered EVs (ITGA5-EVs^-148a^). These engineered EVs were highly effective in targeting pancreatic CAFs, modulating their bioactivity, and consequently suppressing the progression of pancreatic cancer in vitro and in vivo (Fig. 1).

## Materials and Methods

### Tissue sample collection

Tissues were obtained from patients who underwent surgical treatment at the Affiliated Hospital of Nantong University. The study protocol was approved by the clinical ethics committee of the Affiliated Hospital of Nantong University (2021-L111) and performed according to the guidelines of the Declaration of Helsinki. All samples were pathologically identified as PDAC samples, which were obtained from patients who had not undergone radiotherapy or neoadjuvant chemotherapy.

### CAF isolation and identification

Pancreatic CAFs and NFs were isolated using the outgrowth method. Fresh pancreatic cancer tissues and para-cancer tissues were sliced into pieces and cultured in Dulbecco’s modified Eagle’s medium (DMEM) (Gibco) supplemented with 10% fetal bovine serum (FBS) (Gibco) and 1% penicillin–streptomycin (Gibco). CAFs and NFs were identified by detecting the CAF-specific markers ACTA2, FAP, and FSP using immunofluorescence analysis (Fig. S1A).

### BMSC isolation and identification

Bone marrow samples were obtained from a patient with bone fracture after obtaining informed consent under aseptic conditions. The bone marrow (5 to 10 ml) was extracted from the fracture end with a 20-ml syringe and rapidly mixed with heparin. Next, the bone marrow was diluted with phosphate-buffered saline (PBS) (1:1) and centrifuged in a centrifuge tube containing the same volume of Ficoll lymphocyte separation fluid at 2,000 rpm for 20 min. The nuclear cells were collected from the interface with a straw and washed twice with PBS. Cells were cultured in DMEM (Gibco) supplemented with 10% FBS (Gibco) and 1% penicillin–streptomycin (Gibco). BMSCs at passage 3 were detached and seeded in 6-well plates. The adipogenic, osteogenic, and chondrogenic differentiation of human BMSCs was identified using human BMSC differentiation medium kit (Cyagen) (Fig. S1B). Flow cytometric analysis was used to detect surface markers CD11B, CD14, CD29, CD34, CD45, CD73, and HLA-DR of BMSCs with human MSC surface marker detection kit (Cyagen) (Fig. S1C).

### Cell culture and coculture

The human pancreatic cancer cell line PANC-1 was obtained from Procell Life Science&Technology Co. Ltd. PANC-1 was cultured with DMEM (Gibco) supplemented with 10% FBS (Gibco) and 1% penicillin–streptomycin (Gibco). Cells were passaged using 0.25% trypsin-EDTA (Biochannel) and stored at −80°C in a serum-free cell-freezing medium (New cell & Molecular Biotech Co. Ltd.). For BMSC and CAF coculture, 0.4-μm transwell chambers (Biofil) were used. CAFs were implanted into the lower chamber, and BMSCs were implanted into the upper chamber. GW4869 (MCE) was added into the upper chamber at a concentration of 10 μM. GW4869 is an inhibitor of neutral sphingomyelinase, which reduces the production of ceramide and subsequently inhibits exosome release.

### Bioinformatics analysis

Pancreatic cancer gene expression datasets were retrieved from the Gene Expression Omnibus (GEO) database (https://www.ncbi.nlm.nih.gov/geo/). Six GEO datasets (GSE25820, GSE28955, GSE43796, GSE60980, GSE125538, and GSE163031) were used to identify differentially expressed genes in pancreatic cancer with the R package “limma” based on the following criteria: |logFC| > 1; *P* < 0.05. The results were visualized with the R package “ggplot2.” The OS and DSS in TCGA-pancreatic cancer cohort were analyzed using the R packages “survival” and “survminer.” ROC analysis of ITGA5 was performed using the R package “pROC.”

### Cell transfection

The sequences of the miR-148a-3p mimic or inhibitor were designed and synthesized by GenePharma (Suzhou, China). The si-ITGA5 sequences were designed and synthesized by RiboBio (Guangzhou, China). The ITGA5 overexpression plasmid was designed and synthesized by Miaolingbio (Wuhan, China). Cell transfection was performed when the cells reached 60% to 80% confluency. All transfections were performed using Lipo800 reagents (Beyotime Institute of Biotechnology, Jiangsu, China), following the manufacturer’s instructions.

### Reverse transcription-quantitative polymerase chain reaction

Total RNA was extracted with TRIzol reagent (Invitrogen, USA). cDNA was reverse transcribed using the SweScript RT I First Strand cDNA Synthesis Kit (Servicebio, China). Polymerase chain reaction (PCR) amplification was performed using the SYBR Green qPCR Master Mix (Servicebio, China) in the Applied Biosystems StepOne system. The primers for miR-148a-3p and U6 were obtained from RiboBio. The primers for ITGA5, TERT, and GAPDH were obtained from Generay (Shanghai, China). The relative quantification of gene expression was performed using the 2^−ΔΔCt^ method.

### Western blot

Cells or EVs were lysed with radioimmunoprecipitation assay buffer (Solarbio, China) containing protease inhibitors and phosphatase inhibitors and quantified by bicinchoninic acid (BCA) analysis (Beyotime, China). Then, protein extractions were separated by SDS-PAGE, transferred onto polyvinylidene fluoride (PVDF) membranes (Millipore Corporation, USA), and incubated with the following primary antibodies at 4°C: ACTA2 (1:1,000, Servicebio), FAP (1:500, Beyotime), FSP (1:500, Proteintech), ITGA5 (1:500, Proteintech), TGFBR1 (1:500, Servicebio), p-SMAD2/3 (1:1,000, Abcam), SMAD2/3 (1:500, Servicebio), CD9 (1:1,000, Abcam), CD63 (1:1,000, Abcam), CD81 (1:1,000, Abcam), Alix (1:1,000, Abcam), Tsg101 (1:1,000, Abcam), calnexin (1:1,000, Wanleibio), and GAPDH (1:1,000, Servicebio). Subsequently, the membranes were incubated with secondary antibodies (1:10,000, Servicebio) for 60 min at room temperature. Bands were detected by a chemiluminescence imaging system (Tanon, China).

### 5-Ethynyl-2′-deoxyuridine staining

Cells were seeded into slides in 24-well plates and incubated with culture medium containing 10 μM 5-ethynyl-2′-deoxyuridine (EdU) (Beyotime) at 37°C with 5% CO_2_ for 2 h (PC cells) or 3 h (CAFs and BMSCs). Slides were washed with PBS twice, fixed with 4% paraformaldehyde for 10 min, and permeabilized by 0.5% Triton X-100 for 10 min. Then, slides were incubated with Click Additive Solution containing Azide 594 and CuSO_4_ for 30 min at room temperature. Next, the slides were incubated with Hoechst 33342 for 10 min. Images were captured under a fluorescence microscope (Olympus).

### Transwell and wound-healing assays

For transwell migration assays, 8-μm transwell chambers (Biofil) were used. Cells were seeded into the upper chamber with FBS-free medium. Medium (600 μl) with 10% FBS was added into the lower chamber. For wound-healing assays, cells were seed into 12-well plates. A pipette tip was used to generate a straight wound in the plate. Then, cells were cultured in FBS-free medium for 24 h. At least 5 fields of view were randomly selected in each well for imaging under microscope.

### Bioinformatic analysis of target gene and dual-luciferase assay

TargetScan, miRtarbase, miRmap, PICTAR, microT, and mirDIP databases were used to predict the target genes of miR-148a-3p. The ITGA5-3′-UTR-WT and ITGA5-3′-UTR-MUT sequences were cloned into the vector (GenePharma). Cells were cotransfected with miR-148a-3p negative control/mimic and WT/MUT ITGA5 using Lipo800 reagents (Beyotime). At 48 h after transfection, the activities of Renilla and firefly luciferase were determined using a luciferase reporter assay system.

### Lentivirus infection of BMSCs

HTERT-encoding lentivirus was purchased from Genechem, Shanghai, China, while miR-148a-3p-encoding lentivirus was purchased from Corues Biotechnology, Nanjing, China. The primary BMSCs were seeded in 6-well plates and infected with virus (multiplicity of infection = 100) and HiTransG P (Genechem). At 72 h after infection, BMSCs were cultured in a medium containing 0.5 μg/ml puromycin to amplify the stable cell lines.

### Synthesis and characterization of DSPE-PEG-CRYYRITY

The ITGA5-targeting peptide CRYYRITY was synthesized and identified using high-performance liquid chromatography (HPLC) and mass spectrometry (MS). DSPE-PEG-NHS (1,2-distearoyl-sn-glycero-3-phosphoethanolamine–polyethylene glycol–*N*-hydroxysuccinimide) was purchased from Ruixi Biological Technology (Xi’an, China). The molecular weight of PEG was 2 kDa. DSPE-PEG-NHS (1 eq) and CRYYRITY (1.1 eq) were dissolved in dimethylformamide. Subsequently, the reaction mixture was incubated with triethylamine (3 eq) at room temperature for 12 h. The obtained DSPE-PEG-CRYYRITY was purified by dialyzing for 24 h using a dialysis bag [molecular weight cutoff: 3.5 kDa] and subjected to freeze-drying. Finally, DSPE-PEG-CRYYRITY was dissolved in dimethyl sulfoxide and detected using nuclear magnetic resonance (NMR) spectroscopy.

### Isolation and identification of EVs

To collect the supernatant, the BMSCs were cultured in DMEM/F12 supplemented with 10% exosome-free FBS. FBS was centrifuged for 18 h at 20,0000*g* to remove the EVs. BMSCs were seeded in T175 flasks, and the supernatant was collected after 48 h. The cell supernatant was centrifuged at 300*g* for 10 min, followed by centrifugation at 2,000*g* for 10 min to remove cells and dead cells. Next, the supernatant was centrifuged at 10,000*g* for 30 min to remove cell debris. Further, the supernatant was subjected to ultracentrifugation using an ultracentrifuge (Beckman) at 200,000*g* for 70 min. All the centrifugation steps were performed at 4°C. EVs were resuspended in sterile PBS and stored at −80°C for later use. Protein concentration of EVs was determined using the BCA detection reagent (Beyotime). The collected EVs were diluted 400 times for nanoparticle tracking analysis (NTA). The diluted EVs were absorbed in a 1-ml syringe and injected into the particle tracking analyzer (Particle Metrix, Germany). To perform transmission electron microscopy (TEM) analysis, EVs were dropped into the carbon-coated copper mesh, stained with uranyl acetate, dried at room temperature, and viewed with a Hitachi TEM system.

### In vitro cellular uptake of EVs

EVs were labeled with DiD (Beyotime), following the manufacturer’s instructions. To examine the internalization of EVs using fluorescence microscopy, PANC-1 cells and CAFs were seeded into the wells of 24-well chamber slides and cultured in DMEM supplemented with exosome-depleted 10% FBS for 24 h and incubated with EVs for 8 h. The samples were washed with PBS, stained with 4′,6-diamidino-2-phenylindole (DAPI), and imaged using a confocal microscope (Olympus FV1000).

### Stromal-rich xenograft tumor models

Animal experiments were approved by the Animal Ethics Committee of Southeast University (20220812018). BALB/c male nude mice (aged 4 to 6 weeks; Hangzhou Ziyuan Laboratory Animal Technology Co. Ltd., Hangzhou, China) were housed in a specific pathogen-free laboratory under the following conditions: circadian cycle, 12-h light/dark cycle; access to food and water, ad libitum. The mice were allowed to acclimatize for 1 week before the experiment. To establish stomal-rich tumors, 4 × 10^6^ PANC-1 cells and 1 × 10^6^ CAFs were mixed in the ratio 1:1 in 100 μl of ice-cold Matrigel (Corning) and subcutaneously injected into the left flank of the nude mouse. The tumor size was monitored once every 3 days. Tumor volume was calculated as follows: Volume = (length × width^2^)/2.

### Analysis of EV biodistribution

When the tumor volume reached more than 500 mm^3^, the mice were randomized into 2 groups (*n* = 3). EVs and ITGA5-EVs were labeled with DIR (US EVERBRIGHT), a lipophilic fluorescent dye. EVs (200 μg/mouse) were intravenously injected through the tail vein. Mice were anesthetized and scanned at different time points (0.5, 2, 4, 8, and 12 h) using an IVIS system (PerkinElmer, USA) at 710/780 nm. To determine ex vivo biodistribution, mice were sacrificed and surgically dissected at 12 h. The fluorescence intensities in the heart, liver, spleen, lung, kidney, brain, and tumors were recorded and compared. Additionally, DiD-labeled EVs and ITGA5-EVs were intravenously injected into mice. At 2 h after injection, tumor samples were collected, frozen, sectioned under light, stained with DAPI, and imaged using a confocal microscope (Olympus FV1000) to observe the distribution of EVs in tumors.

### Evaluation of in vivo antitumor efficacy

Mice were randomly divided into the following 4 groups (*n* = 5) when the tumor volume reached more than 100 mm^3^: PBS-treated, ITGA5-EV-treated, (3) EVs^-148a^-treated, and ITGA5-EVs^-148a^-treated groups. EVs (100 μg/ mouse) were injected through the tail vein once every 3 days 6 times. Tumor size was measured using a vernier caliper once every 3 days. At the end of treatment, the mice were anesthetized and sacrificed. Tumor weight was measured at the end of treatment. Tumors and major organs were harvested for subsequent analysis. The tumor samples were fixed with 4% paraformaldehyde and sectioned for immunofluorescence or immunohistochemical staining of ITGA5, ACTA2, FSP, and PCNA. The heart, liver, spleen, lung, and kidney were subjected to hematoxylin and eosin (HE) staining to evaluate histological changes.

### Statistical analysis

All statistical analyses were performed using GraphPad Prism software 8.0. Data are presented as mean ± SD. Data were analyzed using Student’s *t* test between 2 groups and one-way or 2-way analysis of variance (ANOVA) multiple comparison test for multiple groups. Differences were considered significant at *P* < 0.05 (*P < 0.05, **P < 0.01, and ***P < 0.001).

## Results

### miR-148a-3p expression in pancreatic cancer and CAFs

**Fig. 2.**
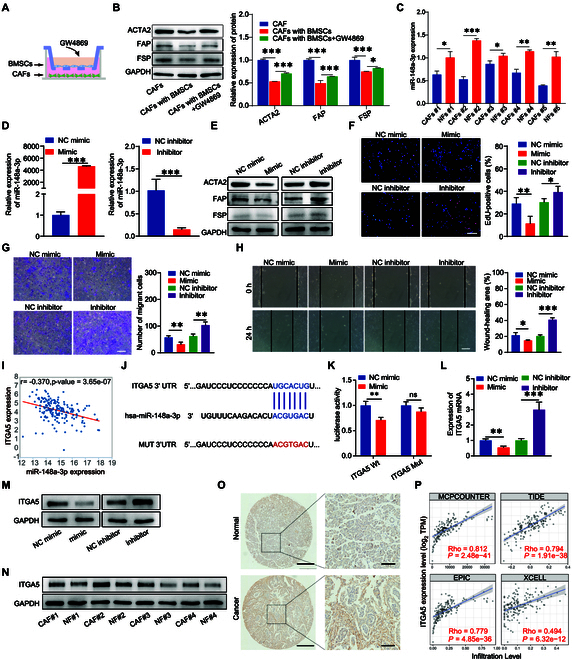
The role of miR-148a-3p in pancreatic CAFs and the expression of downstream target gene ITGA5. (A and B) The activation markers of CAFs were down-regulated upon coculturing CAFs with BMSCs but were up-regulated upon treatment with GW4869 (*n* = 3). (C) qRT-PCR analysis of miR-148a-3p expression in cultured primary CAFs and normal fibroblasts (NFs) (*n* = 3). (D) qRT-PCR analysis revealed the transfection efficiency of miR-148a-3p mimic and inhibitor (*n* = 3). (E) Western blotting analysis of ACTA2, FAP, and FSP protein expression levels in CAFs transfected with miR-148a-3p mimic and inhibitor. (F) EdU assay results of miR-148a-3p-transfected pancreatic CAFs (*n* = 4). (G) Transwell migration assay results of miR-148a-3p-transfected pancreatic CAFs (*n* = 4). (H) Wound-healing assay results of miR-148a-3p-transfected pancreatic CAFs (*n* = 4). (I) Correlation between miR-148a-3p and ITGA5 expression levels in TCGA-pancreatic cancer cohort. (J) Predicted binding site between miR-148a-3p and ITGA5. (K) Dual-luciferase reporter assay results of miR-148a-3p and ITGA5 (*n* = 3). (L) Effect of miR-148a-3p transfection on *ITGA5* mRNA expression (*n* = 3). (M) Effect of miR-148a-3p transfection on ITGA5 protein expression. (N) ITGA5 protein expression levels in primary cultured CAFs and NFs. (O) TMA of *ITGA5* expression in pancreatic cancer and adjacent noncancerous tissues. (P) Correlation between ITGA5 expression and CAF infiltration based on 4 algorithms (MCPOUNTER, TIDE, EPIC, and XCELL). *P < 0.05, **P < 0.01, ***P < 0.001 for all statistical data. Scale bar, 100 μm for all captured pictures.

Several studies, both preclinical and clinical, have shown that BMSCs can alleviate fibrosis progression in different organs by impacting fibroblasts [15,26,27]. Through coculture experiments involving BMSCs and CAFs, we observed that BMSCs may inhibit the activation of CAFs. However, this process can be reversed by the addition of GW4869, an inhibitor of neutral sphingomyelinase that inhibits exosome release (Fig. 2A and B). Bioinformatics analysis revealed that the expression level of miR-148a-3p was highly enriched in BMSC-derived EVs (Fig. S1D), while the miR-148a-3p expression level in pancreatic cancer tissues was lower than that in healthy tissues (criterion |log fold change (FC)| > 1 and *P* < 0.05; Fig. S2A). Additionally, the overall survival (OS) and disease-specific survival (DSS) of patients with pancreatic cancer exhibiting up-regulated miR-148a-3p expression were higher than those of patients with pancreatic cancer exhibiting down-regulated miR-148a-3p expression (Fig. S2B). The expression level of miR-148a-3p had a moderate negative correlation with ACTA2 and FAP, and a weak negative correlation with FSP (Fig. S2C). Quantitative real-time PCR (qRT-PCR) analysis confirmed that miR-148a-3p expression was down-regulated in various primary CAFs (Fig. 2C).

**Fig. 3.**
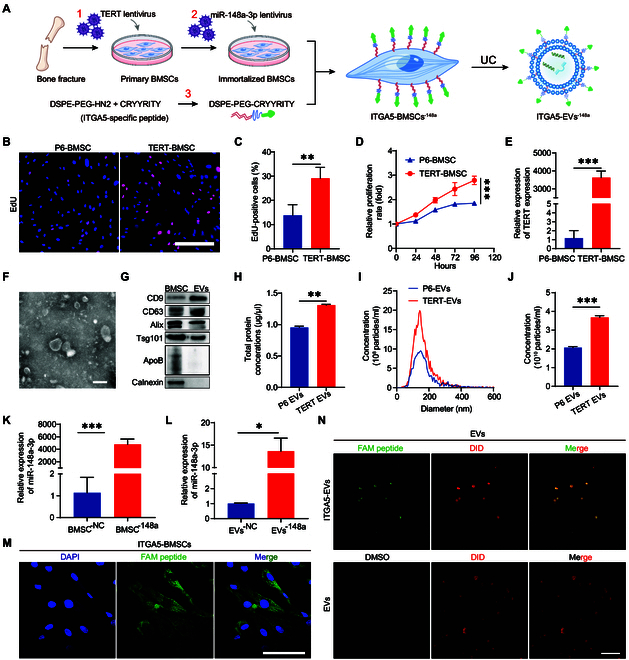
Design and establishment of genetically engineered ITGA5-EVs^-148a^. (A) Schematic diagram illustrating the process of constructing genetically engineered EVs. (B) Proliferation of P6 primary BMSCs and TERT-BMSCs. Scale bar, 100 μm. (C) EdU assay results of P6 primary BMSCs and TERT-BMSCs (*n* = 3). (D) CCK-8 assay results of P6 primary BMSCs and TERT-BMSCs (*n* = 3). (E) qRT-PCR analysis of *TERT* mRNA expression levels in P6 primary BMSCs and TERT-BMSCs (*n* = 3). (F) TEM analysis of TERT-BMSC-EVs. Scale bar, 100 nm. (G) Western blotting analysis of EV markers in EVs. The BMSC lysate served as a control. (H) Qualification of protein concertation in EVs using the BCA assay (*n* = 3). (I) NTA of the size distributions of EVs. (J) NTA analysis of the particle numbers of EVs. (K) qRT-PCR analysis of miR-148a-3p expression in BMSCs (*n* = 3). (L) qRT-PCR analysis of miR-148a-3p expression in EVs (*n* = 3). (M) Laser scanning confocal microscopy (LSCM) confirmed the incorporation of DSPE-PEG-CRYYRITY into the cell membrane of BMSCs. Scale bar, 100 μm. (N) LSCM confirmed the incorporation of DSPE-PEG-CRYYRITY into EVs. Scale bar, 1 μm. *P < 0.05, **P < 0.01, ***P < 0.001 for all statistical data.

### miR-148a-3p interacted with ITGA5 to inactivate pancreatic CAFs

To examine the effect of miR-148a-3p on the biological properties of CAFs and identify potential membrane targets for CAF targeting, miR-148a-3p was overexpressed or knocked down in CAF cells. The transfection efficiency was determined using qRT-PCR analysis. miR-148a-3p expression was up-regulated in the miR-148a-3p mimic-transfected groups and down-regulated in the miR-148a-3p inhibitor-transfected groups (Fig. 2D). The protein levels of ACTA2, FAP, and FSP were examined in CAFs transfected with miR-148a-3p mimic or inhibitor. The ACTA2, FAP, and FSP levels were down-regulated in miR-148a-3p mimic-transfected CAFs and up-regulated in miR-148a-3p inhibitor-transfected CAFs (Fig. 2E and Fig. S3A and B). The results of the proliferation, transwell, and wound-healing assays demonstrated that cell proliferation and migration were down-regulated in miR-148a-3p mimic-transfected CAFs and up-regulated in miR-148a-3p inhibitor-transfected CAFs (Fig. 2F to H). These results demonstrated that miR-148a-3p overexpression inactivates pancreatic CAFs in vitro.

Next, the downstream target genes of miR-148a-3p were predicted using TargetScan, miRtarbase, miRmap, PICTAR, microT, and mirDIP databases, which revealed 802, 403, 442, 323, 949, and 220 potential genes, respectively (Fig. S3C). Among these candidates, *ITGA5* expression was substantially and negatively correlated with miR-148a-3p expression in pancreatic cancer (Fig. 2I). To verify if *ITGA5* was the target gene of miR-148a-3p, a specific binding site between miR-148a-3p and ITGA5 was predicted (Fig. 2J). The dual-luciferase reporter assay results demonstrated that miR-148a-3p mimic transfection decreased the luciferase activity of wild-type (WT) miR-148a-3p-binding site in the 3′-untranslated region (UTR) of *ITGA5* (ITGA5-3′-UTR-WT) (*P* < 0.05) but did not significantly affect the luciferase activity of mutant (MUT) miR-148a-3p-binding site in the 3′-UTR of ITGA5 (ITGA5-3′-UTR-MUT) (*P* > 0.05) (Fig. 2K). Additionally, CAFs were transfected with a miR-148a-3p mimic inhibitor. qRT-PCR and Western blotting analysis revealed that miR-148a-3p mimic down-regulated the ITGA5 mRNA (Fig. 2L) and protein expression levels (Fig. 2M and Fig. S3D). In contrast, miR-148a-3p inhibitor up-regulated the ITGA5 mRNA and protein levels. These results suggest that miR-148a-3p specifically targets and regulates ITGA5 expression. Besides, Western blotting analysis revealed that ITGA5 expression in pancreatic CAFs was up-regulated when compared with that in control fibroblasts (NFs) (Fig. 2N and Fig. S3E)

Additionally, ITGA5 expression in pancreatic cancer tissues was higher than that in noncancerous tissues (Fig. S3F). ITGA5 expression was positively correlated with T stage, N stage, pathological stage, and histologic grade (Fig. S3G to J). The receiver operating characteristic (ROC) curve revealed that ITGA5 expression can distinguish patients with cancer from those without cancer [area under the curve (AUC) = 0.944; confidence interval (CI): 0.919 to 0.970] (Fig. S3K). Based on the time-dependent ROC curve, the AUC values for predicting 1-, 3-, and 5-year survival were 0.568, 0.614, and 0.712, respectively (Fig. S3L). Logistic regression analysis demonstrated that ITGA5 expression was closely associated with various clinical characteristics associated with poor prognosis (pathological stage, T stage, N stage, and histological grade) (Table 1). Immunohistochemical analysis of tissue microarray (TMA) revealed that ITGA5 expression in pancreatic cancer tissues was higher than that in adjacent noncancerous tissues (Fig. 2O). Based on the ITGA5 pathological score in TMA, patients were divided into high-expression and low-expression groups. Analysis of the correlation between the pathological score of ITGA5 and the clinical information of patients with pancreatic cancer revealed that ITGA5 was associated with the pathological stage and T stage of pancreatic cancer (Table 2). Bioinformatics analysis with 4 algorithms confirmed that ITGA5 expression was positively correlated with CAF infiltration in pancreatic cancer (Fig. 2P). As ITGA5 expression was up-regulated on the CAF surface, ITGA5 can be a potential candidate for developing CAF-targeting strategies.

**Table 1.** Logistic regression of ITGA5 in TCGA pancreatic cancer patients.

Characteristics	Total(N)	Odds Ratio(OR)	P value
Gender (Female vs. Male)	178	1.255 (0.925-1.703)	0.144
Pathologic stage (Stage II&Stage III&Stage IV vs. Stage I)	175	1.764 (1.120-2.778)	0.014*
T stage (T3&T4 vs. T1&T2)	176	1.618 (1.091-2.399)	0.017*
N stage (N1 vs. N0)	173	1.561 (1.105-2.205)	0.012*
M stage (M1 vs. M0)	84	0.849 (0.313-2.298)	0.747
Histologic grade (G3&G4&G2 vs. G1)	176	1.722 (1.144-2.593)	0.009**

*P < 0.05, **P < 0.01. Bold indicates statistical differences.

**Table 2.** Correlation of ITGA5 pathological scores with clinicopathological parameters based on tissue microarrays.

**Characteristics**	ITGA5 pathological score		*P* value
	Low (34)	High (32)	
Gender			
Female	18 (27.3%)	15 (22.7%)	0.805
Male	16 (24.2%)	17 (25.8%)	
Age			
<60	11 (16.7%)	10 (15.2%)	1.000
≥60	23 (34.8%)	22 (33.3%)	
Pathologic stage			
I	15 (22.7%)	7 (10.6%)	0.038*
II	18 (27.3%)	19 (28.8%)	
III-IV	1 (1.5%)	6 (9.1%)	
T stage			
T1	10 (15.2%)	1 (1.5%)	0.002**
T2	21 (31.8%)	19 (28.8%)	
T3	3 (4.5%)	10 (15.2%)	
T4	0 (0%)	2 (3%)	
N stage			
0	18 (29.5%)	9 (14.8%)	0.134
1	15 (24.6%)	19 (31.1%)	

*P < 0.05, **P < 0.01. Bold indicates statistical differences.

### Design and establishment of ITGA5-EVs^-148a^

To obtain sufficient EVs from BMSCs for treatment, primary BMSCs were engineered with TERT-encoding lentivirus to establish an immortalized BMSC line for EV production (Fig. 3A1). Primary BMSCs can be subcultured for several generations, yielding flattened or aggregated cells (senescent cells) after passage 6 (Fig. S4A). Green fluorescent protein (GFP) fluorescence demonstrated the success of BMSC infection (Fig. S4B). The immortalized BMSC cell line (Fig. S4C) exhibited a spindle shape at passage 3. The results of the EdU and cell counting kit-8 (CCK-8) assays demonstrated that the proliferation rate of immortalized BMSCs was higher than that of P6 BMSCs (Fig. 3B to D). The cellular *TERT* mRNA expression levels were examined using qRT-PCR analysis. The *TERT* mRNA levels in immortalized BMSCs were significantly higher than those in P6 BMSCs (Fig. 3E). These results demonstrate the successful establishment of immortalized BMSC lines with enhanced proliferation capacity.

**Fig. 4.**
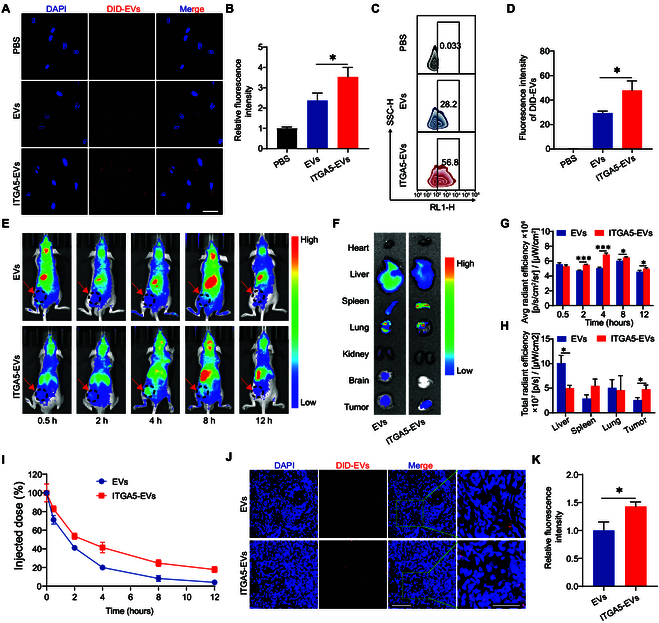
ITGA5-targeting peptide enhanced the targeting ability of EVs. (A) Internalization of EVs and ITGA5-EVs by CAFs. Scale bar, 100 μm. (B) Qualification of the fluorescence intensity of CAFs incubated with different EVs using LSCM. (C) Flow cytometric analysis of CAFs incubated with different EVs (*n* = 3). (D) Qualification of the fluorescence intensity of CAFs incubated with different EVs using flow cytometry. (E) In vivo fluorescence images of tumor-bearing mice treated with different EVs or ITGA5-EVs at different time points (*n* = 3). (F) Ex vivo fluorescence images of major organs and tumor tissues at 12 h after EV administration. (G) Qualification of the fluorescence intensity in tumor sites at different time points (*n* = 3). (H) Qualification of the fluorescence intensity of major organs at 12 h after EV administration (*n* = 3). (I) Peripheral blood fluorescence intensity after EV administration. (J) Fluorescence images of DiD-labeled EVs and ITGA5-EVs targeting and penetrating the tumor observed using LSCM. Scale bars, 100 μm (left) and 50 μm (right). (K) Qualification of DiD-labeled EVs and ITGA5-EVs in tumors (*n* = 3). *P < 0.05, ***P < 0.001 for all statistical data.

Next, the EVs were extracted from the cell culture supernatant using ultracentrifugation. TEM revealed that the EVs exhibited a spherical morphology with a phospholipid bilayer membrane (Fig. 3F). Western blotting analysis revealed the expression of the biomarker proteins CD9, CD63, CD81, Alix, and TSG101, but not that of calnexin, in the EVs (Fig. 3G). BCA protein assay results indicated that the protein levels in TERT-BMSC-derived EVs were up-regulated when compared with those in primary P6 BMSCs (Fig. 3H). Additionally, NTA revealed that the average diameter of BMSCs EVs was in the range of 130 to 140 nm (Fig. 3I), which was within the known diameter range of EVs (50 to 200 nm). Furthermore, NTA revealed that the number of EVs from TERT-BMSCs was higher than that of EVs from primary P6 BMSCs (Fig. 3J). Next, miR-148a-3p-encoding lentivirus was used to infect immortalized BMSC line to establish an immortalized miR-148a-3p-overexpressing BMSC line (Fig. 3A2). qRT-PCR analysis revealed that miR-148a-3p-encoding lentivirus transduction significantly up-regulated miR-148a-3p expression (Fig. 3K). In particular, miR-148a-3p expression was up-regulated in EVs (Fig. 3L). This indicates that we have successfully engineered immortalized BMSCs that have a high expression of 148a-3p. This leads to an increased production of EVs with enhanced miR-148a-3p.

As pancreatic CAFs exhibited ITGA5 up-regulation, ITGA5 was selected for modification on the EV surface. ITGA5-targeting peptide CRYYRITY (Fig. S4D) was first conjugated to DSPE-PEG-NHS to generate DSPE-PEG-CRYYRITY (Fig. S4E). The results of HPLC and MS analyses of CRYYRITY are shown in Fig. S4F and G. The NMR spectrum of DSPE-PEG-CRYYRITY is shown in Fig. S4H. To visually trace the ITGA5-targeting peptide, we constructed DSPE-PEG-CRYYRITY(FAM). DSPE can be easily incorporated into the lipid bilayer of the cell membrane. ITGA5-targeting peptide-modified BMSCs were observed under a confocal microscope. Green fluorescence was observed on the BMSC surface, indicating that DSPE-PEG-CRYYRITY(FAM) was inserted into the cell membrane and that it was used for the subsequent production of engineered EVs (Fig. 3M). To verify that the secreted EVs can maintain the inserted DSPE-PEG-CRYYRITY, EVs from engineered BMSCs were labeled with DiD and observed under a confocal microscope. Obvious green fluorescence was observed in the ITGA5-EV group. On the contrary, no green signal was observed in the EV group (Fig. 3N). These results suggest the successful construction of BMSC-derived EVs with miR-148a-3p up-regulation and ITGA5-targeting ability.

### Targeting ability of ITGA5-EVs in vitro and in vivo

To verify the targeting ability of ITGA5-EVs, DiD-labeled EVs and ITGA5-EVs were incubated with CAFs for 10 h. The cellular uptake of DiD-labeled EVs and ITGA5-EVs was compared using confocal microscopy (Fig. 4A). The CAF cytosolic accumulation of ITGA5-EVs (2.37 ± 0.37) was significantly higher than that of EVs (3.53 ± 0.47) (Fig. 4B). Consistently, flow cytometric analysis revealed that the uptake of ITGA5-EVs (29.4 ± 1.59) was higher than that of EVs (47.8 ± 7.9) in pancreatic CAFs (Fig. 4C and D). This can be attributed to the interaction of ITGA5 on the cell surface with the ITGA5-targeting peptide on the engineered EV surface, which was hypothesized to occur during the internalization through receptor-mediated endocytosis. To examine the biodistribution of ITGA5-EVs, the stromal-rich xenograft nude mice model bearing PANC-1 cell-derived tumors and CAFs was used. The dynamic distribution of DIR-labeled EVs was tracked using an in vivo imaging system (IVIS) based on a timeline (Fig. 4E). The fluorescence intensity in mice steadily increased within 4 h and gradually decreased thereafter. ITGA5-EVs exhibited enhanced targeting ability and accumulation at the tumor site. The peak fluorescence signal value in the ITGA5-EV group was higher than that in the EV group (Fig. 4E and G).

**Fig. 5.**
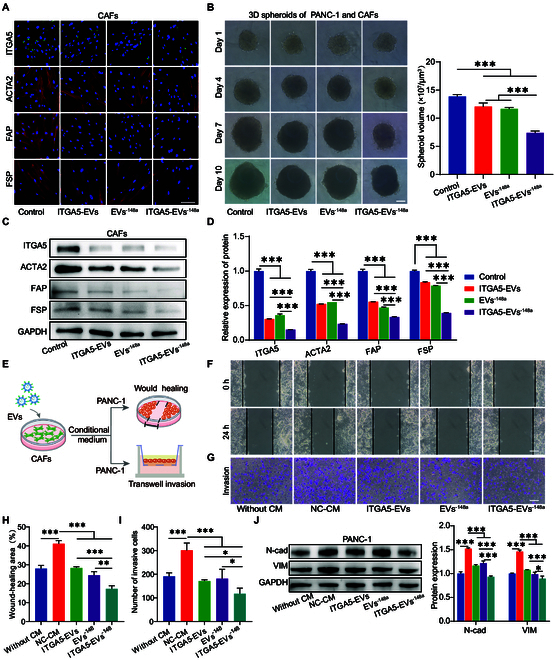
Function of ITGA5-EVs^-148a^ in pancreatic CAFs. (A) Immunofluorescence analysis of ITGA5, ACTA2, FAP, and FSP expression levels in CAFs treated with different EVs using LSCM. (B) Three-dimensional multicellular spheroids treated with different EVs (*n* = 5). (C and D) The protein expression levels of ITGA5, ACTA2, FAP, FSP, TGFBR1, and p-SMAD2/3 in CAFs treated with different EVs were evaluated using Western blotting analysis (*n* = 3). (E) Schematic illustration of the process of obtaining conditional medium (CM) from CAFs treated with different EVs after coculture with PANC-1 cells for evaluating the migration and invasion abilities. (F and H) The migration of PANC-1 cells treated with CAF CM was evaluated using the wound-healing assay (*n* = 3). (G and I) The invasion of PANC-1 cells treated with CAF CM was evaluated using the transwell assay (*n* = 3). (J) Western blotting analysis of N-cad and VIM levels in PANC-1 cells treated with EVs (*n* = 3). *P < 0.05, **P < 0.01, ***P < 0.001 for all statistical data. Scale bar, 100 μm for all captured pictures.

Additionally, the fluorescence signal at the tumor site in the ITGA5-EV group was higher than that in the EV group at 12 h after injection. Major mouse organs were excised and subjected to ex vivo fluorescence imaging (Fig. 4F and H). The hepatic fluorescence signal in the EV group was higher than that in the ITGA5-EV group. Meanwhile, the tumor fluorescence signal in the ITGA5-EV group was higher than that in the EV group. Additionally, the peripheral blood circulation time of ITGA5-EVs was higher than that of EVs (Fig. 4I). Microscopic analysis further demonstrated that the tumor accumulation of ITGA5-EVs was higher than that of EVs (Fig. 4J and K). These results indicate that the targeting efficiency of ITGA5-EVs was higher than that of nonengineered EVs in vitro and in vivo.

### In vitro inhibition of cancer cells through inactivating pancreatic CAFs

To assess the function of ITGA5-EVs^-148a^ in CAFs, CAFs were incubated with PBS, ITGA5-EVs, EVs^-148a^, and ITGA5-EVs^-148a^ for 72 h. Compared with those in the PBS-treated group, the expression levels of ITGA5, ACTA2, FAP, and FSP were significantly down-regulated in the ITGA5-EV-treated and EVs-^148a^-treated groups with the highest inhibitory effect observed in the ITGA5-EVs^-148a^-treated group (Fig. 5A, C, and D). The inactivation of CAFs can weaken the crosstalk between CAFs and cancer cells, suppressing malignancy. Next, the effect of CAF conditioned medium (CM) on the migration and invasion of the pancreatic cancer cell line (PANC-1 cells) was examined (Fig. 5E). The migration ability was assessed using a wound-healing assay. Treatment with CAF CM significantly up-regulated the migration of PANC-1 cells. Pretreatment with EVs significantly mitigated the CAF CM-induced up-regulation of PANC-1 cell migration (Fig. 5F and H). The invasion behavior of PANC-1 cells was examined using the transwell assay. Consistent with the migration assay results, CAF CM promoted the migration of PANC-1 cells to the lower chamber. However, pretreatment with EVs markedly inhibited the invasion of PANC-1 cells (Fig. 5G and I). Western blotting analysis revealed that the expression levels of N-cad and VIM were up-regulated upon treatment with CAF CM. Pretreatment with ITGA5-EVs^-148a^ mitigated the CAF CM-induced up-regulation of E-cad and VIM levels (Fig. 5J). Thus, ITGA5-EVs^-148a^ inactivated CAFs and suppressed the CAF-mediated up-regulation of migration and invasion of cancer cells.

**Fig. 6.**
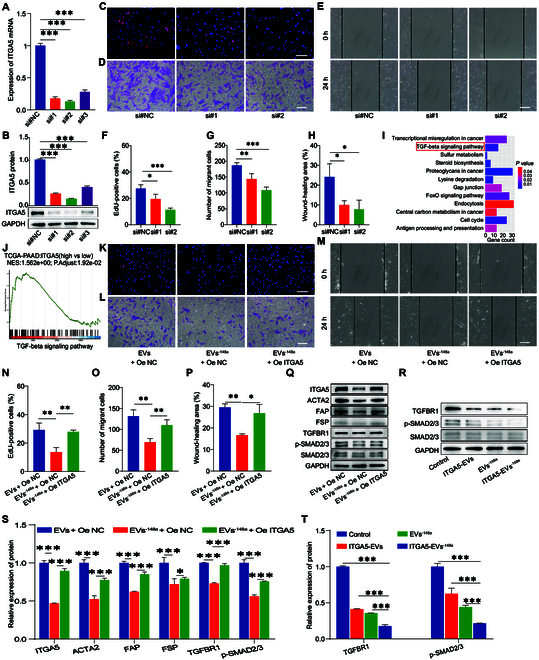
miR-148a-3p/ITGA5 inactivated pancreatic CAFs through the TGF-β/SMAD pathway. (A) The mRNA expression of *ITGA5* in CAFs transfected with short-interfering RNA targeting *ITGA5* (si-ITGA5) was evaluated using qRT-PCR (*n* = 3). (B) The protein expression level of ITGA5 in CAFs transfected with si-ITGA5 was evaluated using Western blotting analysis (*n* = 3). (C to H) The proliferation and migration of CAFs transfected with si-ITGA5 were examined using the EdU (C and F), transwell (D and G), and wound-healing assays (E and H) (*n* = 4). (I) KEGG pathway enrichment (KEGG) analysis of miR-148a-3p. (J) Gene Set Enrichment Analysis (GSEA) of ITGA5. (K to P) The proliferation and migration of CAFs treated with EVs and transfected with ITGA5 overexpression plasmid were analyzed using the EdU (K and N), transwell (L and O), and wound-healing assays (M and P) (*n* = 4). (Q and S) The protein expression levels of ITGA5, ACTA2, FAP, FSP, TGFBR1, and p-SMAD2/3 in CAFs treated with EVs and transfected with ITGA5 overexpression plasmid were examined using Western blotting analysis (*n* = 3). (R and T) Protein expression levels of TGFBR1, p-SMAD2/3, and SMAD2/3 in CAFs treated with different EVs (*n* = 3). *P < 0.05, **P < 0.01, ***P < 0.001 for all statistical data. Scale bar, 100 μm for all captured pictures.

Due to the integrality of ECM assembly and pathophysiological conditions in tumor tissue, multicellular spheroids composed of PANC-1 and CAFs were constructed to evaluate the effect of engineered EVs. When spheroids were formed, EVs were added for further incubation. The results demonstrated that ITGA5-EVs^-148a^ significantly inhibited the growth of 3-dimensional (3D) spheroids (Fig. 5B). These findings suggest that ITGA5-EVs^-148a^ can inhibit the growth of 3D multicellular spheroids of pancreatic cancer.

### ITGA5-EVs^-148a^ reprogrammed pancreatic CAFs by regulating ITGA5 through the TGF-β/SMAD pathway

To elucidate the potential mechanism of ITGA5-EVs^-148a^ in reprograming pancreatic CAFs, *ITGA5* was knocked down in CAFs using ITGA5-specific short-interfering RNA (si-ITGA5). The transfection efficiency was examined using qRT-PCR (Fig. 6A) and Western blotting (Fig. 6B) analyses. Si#1 and si#2 were chosen for subsequent studies. The EdU, transwell, and wound-healing assay results demonstrated that si-ITGA5 suppressed the proliferation and migration of CAFs (Fig. 6C to H). The percent of EdU-positive cells dropped to 71% and 41% compared with si#NC (Fig. 6C and F). The wound-healing ability dropped to 41% and 32% compared with si#NC (Fig. 6E and H). The migration ability dropped to 77% and 58% compared with si#NC (Fig. 6D and G). The data demonstrated that *ITGA5* knockdown suppressed the proliferation and migration of CAFs. Kyoto Encyclopedia of Genes and Genomes (KEGG) pathway enrichment analysis revealed that miR-148a-3p and ITGA5 were enriched in the transforming growth factor-β (TGF-β) signaling pathway (Fig. 6I and J).

**Fig. 7.**
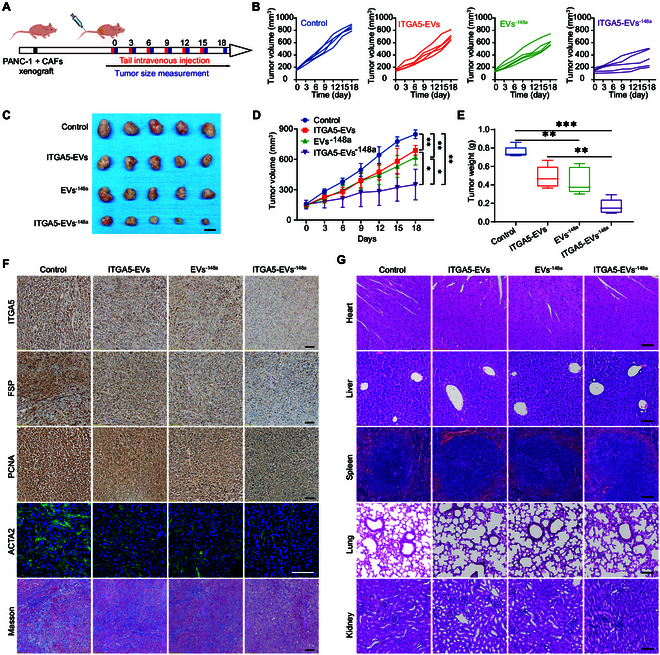
ITGA5-EV^-148a^ reprogrammed CAFs and inhibited tumor growth in vivo. (A) Schematic illustration of animal experiments. (B) Individual tumor growth curve in different groups. (C) Tumors of mice at the end of the treatment. Scale bar, 1 cm. (D) Tumor volume growth curve in different groups (*n* = 5). (E) Tumor weight in different groups at the end of the treatment (*n* = 5). (F) Analysis of ITGA5, FSP, PCNA, and ACTA2 expression levels and Masson staining of tumors in different groups. Scale bar, 100 μm. (G) HE staining of primary mouse organs harvested from different groups. *P < 0.05, **P < 0.01, ***P < 0.001 for all statistical data. Scale bar, 100 μm for all captured pictures.

The role of EVs^-148a^ in suppressing fibrosis progression through ITGA5 was examined using a rescue experiment. ITGA5 overexpression suppressed the EVs^-148a^-induced inhibitory effects on cell proliferation and migration of CAFs (Fig. 6K to P). Western blotting analysis revealed that ITGA5 overexpression mitigated EVs^-148a^-induced ITGA5 down-regulation (Fig. 6Q and S). Additionally, ITGA5 overexpression mitigated the EVs^-148a^-induced down-regulation of TGFBR1, p-SMAD2/3, ACTA2, FAP, and FSP levels (Fig. 6Q and S). The TGFBR1 and p-SMAD2/3 levels in the ITGA5-EVs and EVs^-148a^ groups were down-regulated when compared with those in the PBS group. ITGA5-EVs^-148a^ exerted the strongest inhibitory effect (Fig. 6R and T). These findings suggest that ITGA5-EVs^-148a^ reprograms CAFs through the TGF-β/SMAD pathway.

### In vivo suppression of tumor development

The antitumor efficacy of EVs was evaluated in the stromal-rich xenograft nude mice model bearing PANC-1 and CAFs (Fig. 7A). The tumor volume of ITGA5-EVs and EVs^-148a^ group was significantly suppressed compared with control. The growth-inhibitory effects in the ITGA5-EVs^-148a^-treated group were higher than those in the other groups (Fig. 7B to D). Tumor z

## Discussion

CAFs are known to be the most important cellular component of the ECM [28]. They can remodel the ECM by synthesis and degradation of components to create a dense barrier to defend the immune cell infiltration and create a high interstitial pressure to prevent therapeutic agents [29,30]. CAFs also secrete various soluble mediators that promote tumor proliferation, metastasis, and chemoresistance [31,32]. Thus, it is imperative to find effective therapies for CAF reprogramming. EVs have attracted attention for their therapeutic potential in cancer treatment. Engineered EVs enhance their loading efficiency, targeting ability, and therapeutic effect and thus have great promise to be used as an alternative approach for EV-based therapy [33,34]. This study elucidated the molecular mechanism of miR-148a-3p delivered through BMSC-derived EVs in pancreatic CAFs. Next, engineered ITGA5-EVs^-148a^ was designed to reprogram pancreatic CAFs. In vitro and in vivo experiments demonstrated that engineered ITGA5-EVs^-148a^ exhibited enhanced targeting and pancreatic CAF reprogramming abilities.

Various studies have demonstrated that external BMSC administration exerts effective therapeutic and protective effects on fibrotic diseases [35,36]. Pancreatic cancer is characterized by abundant CAFs in the stroma. The coculture experiment results demonstrated that BMSCs down-regulated the ACTA2, FAP, and FSP protein expression levels in CAFs. Treatment with the EV inhibitor suppressed the inhibitory effects of BMSCs on the ACTA2, FAP, and FSP protein expression levels. Recent studies have demonstrated that EVs are promising natural vectors for gene and drug delivery owing to their low toxicity, low immunogenicity, and high biocompatibility [12]. BMSC-derived EVs exert antitumor effects through multiple mechanisms [13,37]. However, the role of BMSC-derived EVs in the pathological processes of CAFs has not been previously reported.

Analysis of BMSC-derived EVs and pancreatic cancer miRNA array revealed that miR-148a-3p was enriched in BMSC-derived EVs. Additionally, the expression of miR-148a-3p was down-regulated in pancreatic cancer tissues. Furthermore, miR-148a-3p expression was down-regulated in pancreatic CAFs. Previous studies have demonstrated that miR-148a-3p inhibits the progression of lung adenocarcinoma [21], hepatocellular carcinoma [22], and breast cancer [38]. Additionally, miR-148a-3p exerts anti-fibrotic effects in hepatic stellate cells [39,40]. This study demonstrated that miR-148a-3p inhibited the proliferation and migration of CAFs. The Cancer Genome Atlas (TCGA) data analysis indicated that the expression of miR-148a-3p was negatively correlated with that of ACTA2, FAP, and FSP. Western blotting revealed that miR-148a-3p overexpression down-regulated the protein levels of ACTA2, FAP, and FSP in CAFs. Although miRNA sequencing datasets from various databases provide valuable insights, they do not provide useful information on the heterogeneous nature of BMSCs. EVs encompass a spectrum of molecules beyond miR-148a-3p, and the absence of sequencing restricts our understanding of their complete molecular profile. This limitation prompts us to carefully consider and discuss potential unprecedented biological effects or interferences associated with the complex nature of EVs. This study focused on the efficacy of reprogramming technology. However, the effects of diverse EV components are future research focus areas.

Subsequently, we revealed that miR-148a-3p targeted and inhibited ITGA5 (integrin α5). Integrins are a large family of heterodimeric transmembrane glycoproteins consisting of 2 subunits, α and β [41]. Additionally, integrins mediate cell–cell and cell–ECM interactions and play an important role in mechano-transduction, stemness, epithelial plasticity, and therapeutic resistance in cancer [42]. ITGA5 promotes tumor progression through the FAK/AKT pathway [43] and mediates cancer cell–fibroblast adhesion and peritoneal dissemination [44] in gastric cancer. The inhibition of ITGA5 in PSCs suppresses desmoplasia and potentiates the efficacy of chemotherapy in pancreatic cancer [45]. A novel ITGA5 antagonistic peptidomimetic (RYYRITY) was designed, which can specifically bind to ITGA5 and block the downstream signaling. This study suggested that miR-148a-3p negatively regulates ITGA5 through the TGF-β/SMAD pathway. ITGA5 was overexpressed in both pancreatic cancer cells and CAFs. ITGA5 inhibition suppressed the malignancy of cancer cells and attenuated desmoplasia in CAFs.

Primary BMSCs were infected with TERT-encoding lentivirus to construct immortalized BMSCs, which can provide a stable supply of EVs for gene delivery. The immortalized BMSCs were then infected with miR-148a-3p-encoding lentivirus. The EVs from these BMSCs exhibited significantly enhanced miR-148a-3p expression. One study indicated that the peptide iRGD exerts effective therapeutic effects on PDAC with up-regulated ITGB5 expression [46]. This study also synthesized DSPE-PEG-CRYYRITY. The hydrophobic insertion modification method was used to successfully insert DSPE-PEG-CRYYRITY into the cytomembrane of BMSCs. Finally, ITGA5-EVs^-148a^ was constructed. The ITGA5-targeting ability of ITGA5-EVs was higher than that of unmodified EVs. The techniques to immortalize BMSCs through the transduction of TERT-encoding sequences aim to overcome senescence for sustained EV production. However, it is crucial to recognize potential changes in cell behavior and EV content. Previous studies have indicated alterations in immortalized MSCs, such as rapid adenosine triphosphate (ATP) degradation and weakened immune suppression [47]. Conversely, immortalized MSCs can maintain characteristics with potential clinical applications. For example, liver failure can be treated using hTERT-MSC-EVs [48]. This study focused on miR-148a-3p in BMSC-EVs, overlooking potential changes in other bioactive molecules after immortalization. Further studies are needed to investigate the EV components after immortalization. Moreover, we utilized ultracentrifugation for EV isolation, a prevalent method acknowledged for yielding high-purity EVs. However, it may not completely preclude the possibility of lipoprotein contamination. In future investigations, we aim to optimize the EV isolation methodology or implement additional detection modalities to substantiate the heightened purity of the isolated EVs.

To investigate the therapeutical effects of ITGA5-EVs^-148a^ on tumors, the regulatory effects of ITGA5-EVs^-148a^ were evaluated. In vitro experiments demonstrated that ITGA5-EVs^-148a^ significantly down-regulated the expression levels of ITGA5, ACTA2, FAP, and FSP in CAFs. Additionally, ITGA5-EVs^-148a^ inhibited the TGF-β/SMAD signaling pathway. Furthermore, ITGA5-EVs^-148a^ decreased the area of 3D heterospheroids. This study demonstrated that ITGA5-EVs^-148a^ inhibited CAF-mediated paracrine effect on tumor cell migration. These effects of ITGA5-EVs^-148a^ can be attributed to the following 2 mechanisms: ITGA5-specific peptide directly binds to ITGA5 on the cell membrane surface and mediates the downstream signaling; miR-148a-3p specifically inhibits *ITGA5* mRNA expression.

The characteristic size of EVs facilitates their accumulation at cancer sites through the enhanced permeability and retention effect [49]. However, the incorporation of desired ligands on the EV surface can guide the EVs to the target sites where the corresponding cell surface biomarkers are overexpressed. In vivo imaging and ex vivo fluorescence imaging indicated that ITGA5-EVs exhibited enhanced targeting and accumulation at the tumor site.

In vivo experiments demonstrated that ITGA5-EVs^-148a^ significantly suppressed the tumor growth and the expression levels of ITGA5, PCNA, ACTA2, and FSP. However, this study did not incorporate a control group comprising healthy EVs from nonengineered BMSCs in the in vivo experiments. This control group aids in delineating the precise effect of miR-148a-3p overexpression on EV performance. However, comparative analysis between ITGA5-EVs and ITGA5-EVs^-148a^ revealed major distinctions. These findings suggested that the enhanced in vivo efficacy of engineered EVs can be attributed to the overexpression of miR-148a-3p.

## Data Availability

All data generated or analyzed during this study are included in this article.
