# Supplementary material for: Targeting Reprogrammed Cancer-Associated Fibroblasts with Engineered Mesenchymal Stem Cell Extracellular Vesicles for Pancreatic Cancer Treatment
Source: Biomater Res. 2024 Aug 2;28:0050. doi: 10.34133/bmr.0050 (PMC11293949; doi:10.34133/bmr.0050)
Supplement: Supplementary 1 — Figs. S1 to S4 [file bmr.0050.f1.docx]

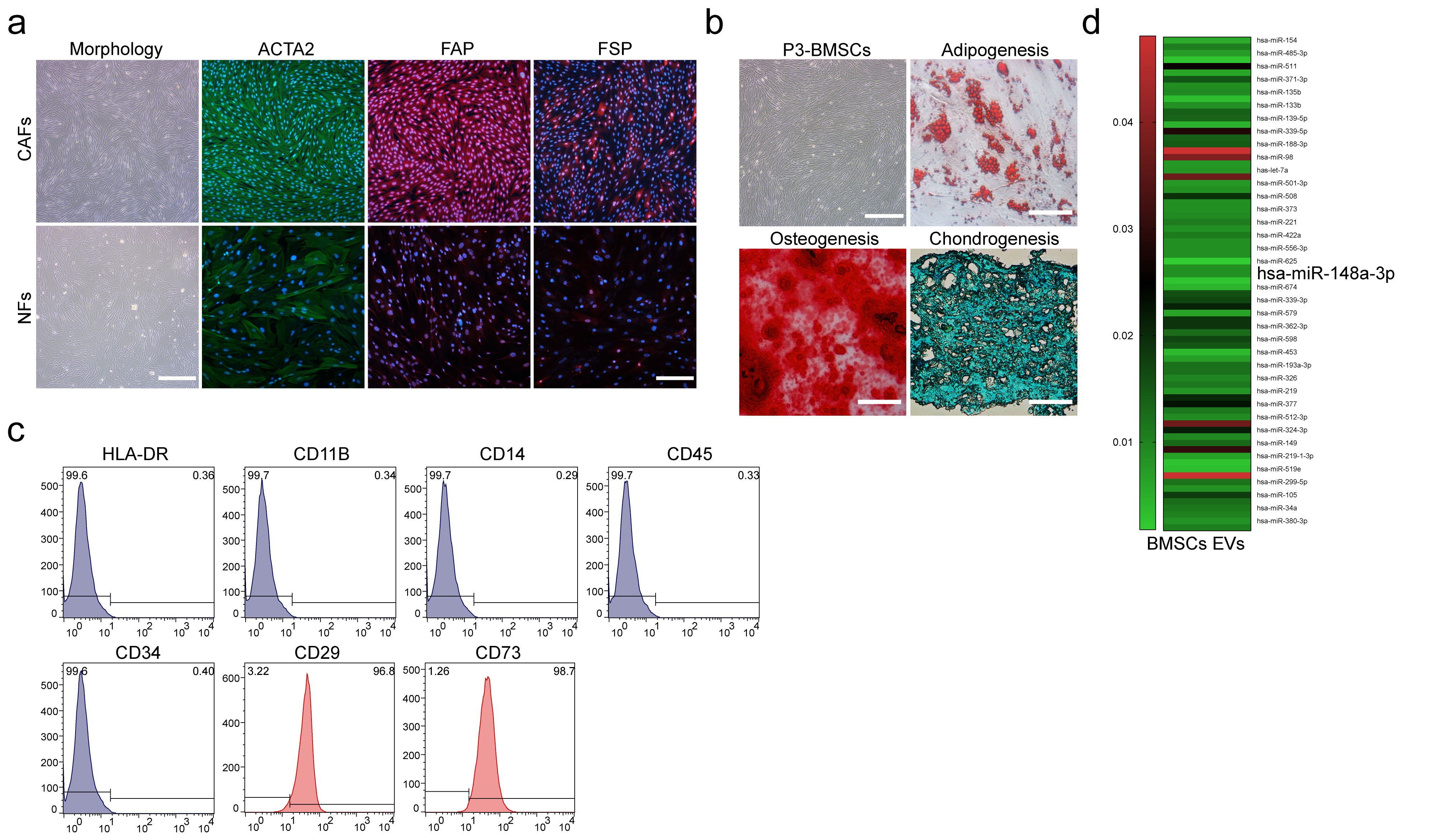
**Supplementary Fig. 1 Primary culture of bone marrow mesenchymal stem cells (BMSCs) and cancer-associated fibroblasts (CAFs). a** Morphology of primary cultured CAFs and healthy fibroblasts (NFs). Immunofluorescence analysis of ACTA2, FAP, and FSP in CAFs and NFs. **b** Morphology of P3 BMSCs and identification of differentiation abilities. **c** Flow cytometric analysis of BMSC markers. (D) MicroRNA (miRNA) array of BMSC-derived extracellular vesicles (EVs). Scale bar = 100 μm for all captured pictures.


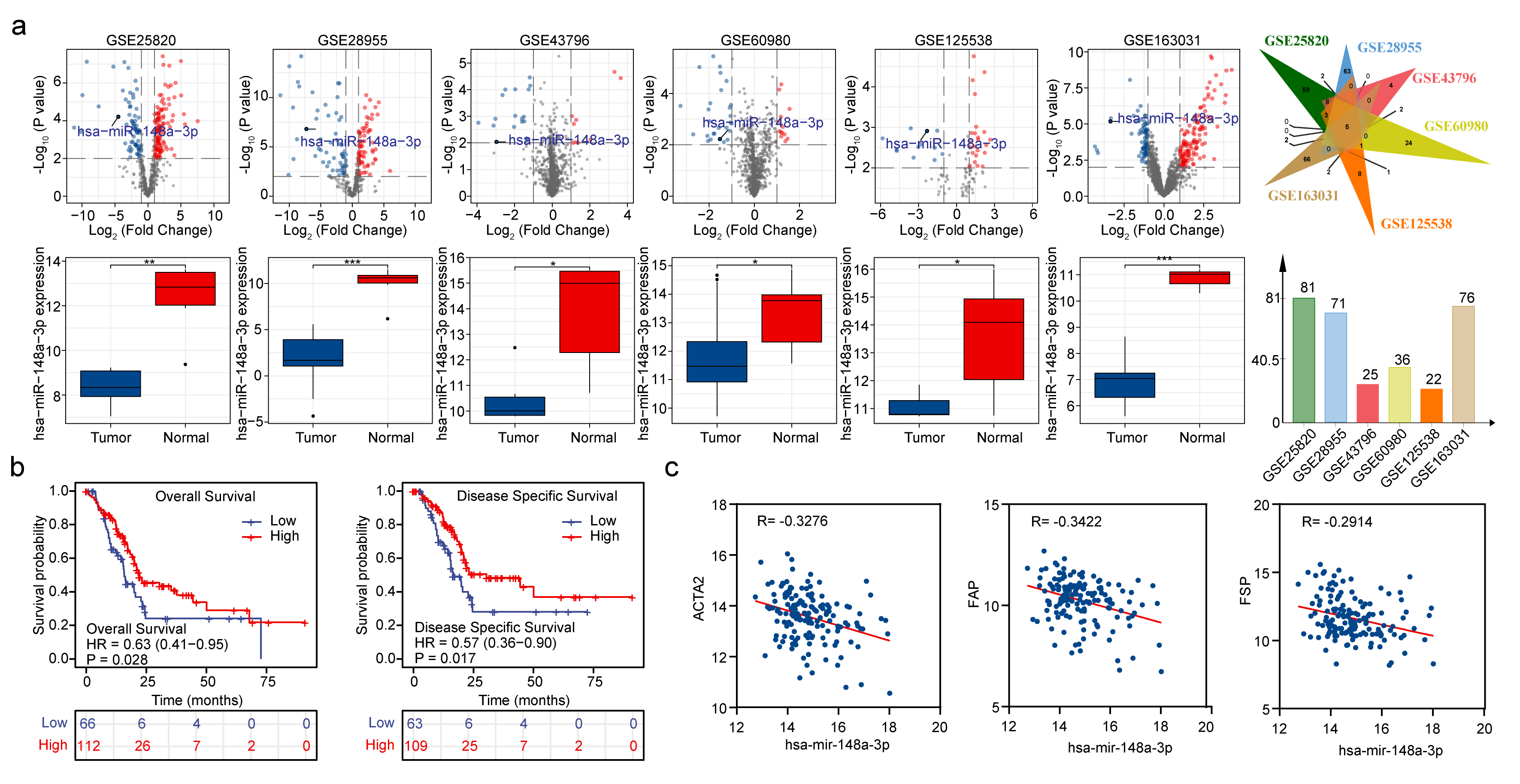


**Supplementary Fig. 2 Expression of miR-148a-3p in pancreatic cancer and pancreatic cancer-associated fibroblasts (CAFs). a** The differential expression profiles of miR-148a-3p between pancreatic cancer and non-cancer tissues (|log fold-change (FC)| >1; p < 0.05). **b** Overall survival (OS) and disease-specific survival (DSS) of patients with pancreatic cancer based on miR-148a-3p expression level. **c** Correlation of the miR-148a-3p expression levels with the ACTA2, FAP, and FSP expression levels in The Cancer Genome Atlas (TCGA)-pancreatic cancer cohort. ^*^p < 0.05, ^**^p < 0.01, ^***^p < 0.001 for all statistical data.


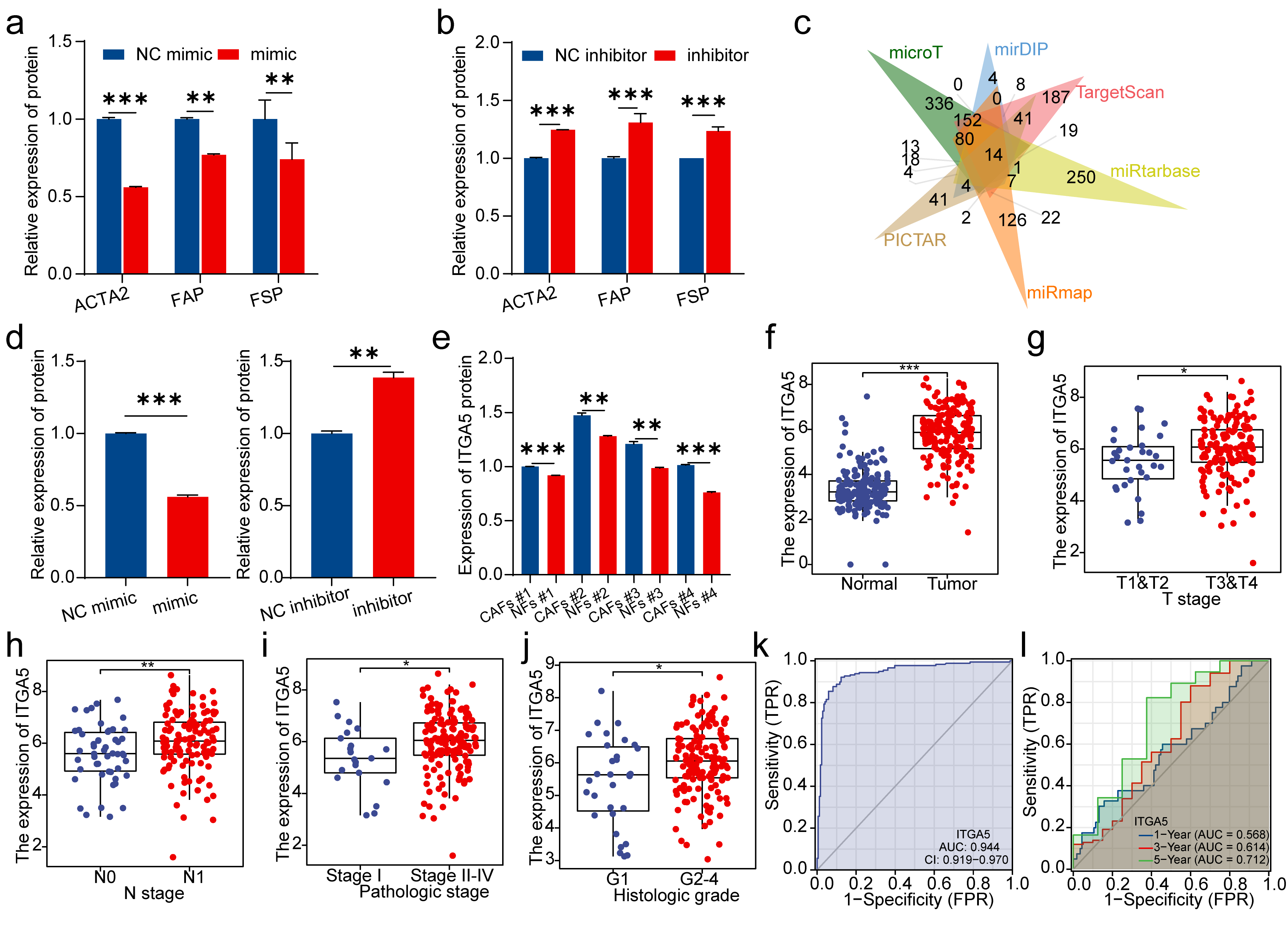
**Supplementary Fig. 3 The role of miR-148a-3p in pancreatic CAFs and correlation between ITGA5 expression and the clinical parameters of The Cancer Genome Atlas (TCGA)-pancreatic cancer cohort. a, b** Quantitative data of western blotting analysis of ACTA2, FAP, and FSP protein expression levels in CAFs transfected with miR-148a-3p mimic and inhibitor (n=3). **c** Venn diagram of miR-148a-3p targets predicted using the TargetScan, miRtarbase, miRmap, PICTAR, microT, and mirDIP databases. **d** Effect of miR-148a-3p transfection on ITGA5 protein expression (n=3). **e** ITGA5 protein expression levels in primary cultured CAFs and normal fibroblasts (NFs) (n=3). **f-j** ITGA5 expression in non-cancerous/cancer tissues, as well as in T stage, N stage, pathologic stage, and histological stage. **k, l** Receiver operating characteristic (ROC) and time-dependent ROC curves of ITGA5 in TCGA-pancreatic cancer cohort. ^*^p < 0.05, ^**^p < 0.01, ^***^p < 0.001 for all statistical data.


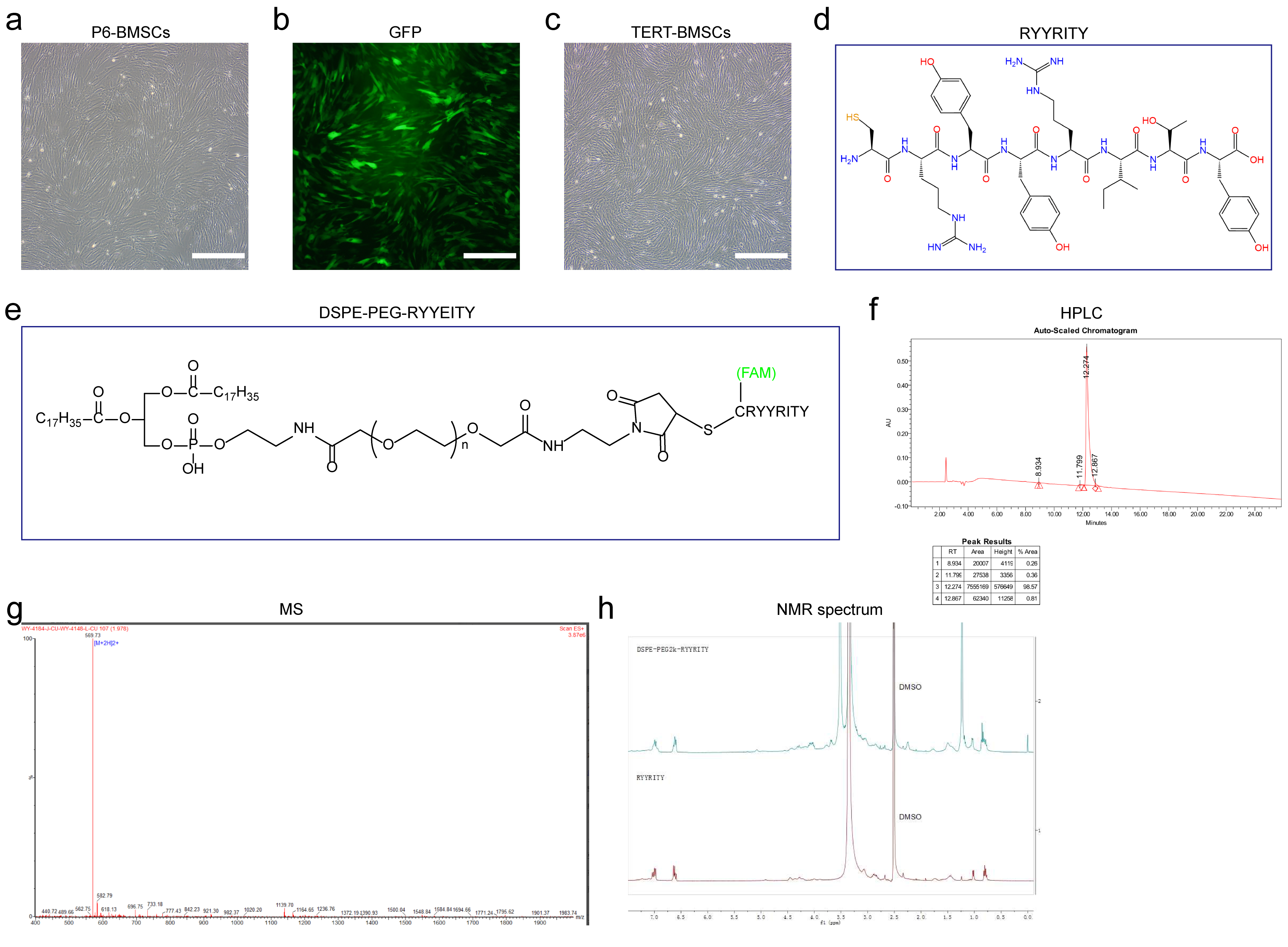
**Supplementary Fig. 4 Engineered bone marrow mesenchymal stem cells (BMSCs) and** **2-distearoyl-sn-glycero-3-phosphoethanolamine-poly (ethylene glycol) (DSPE-PEG)-CRYYRITY.** **a** Morphology of P6-BMSCs. **b** The fluorescence intensity of green fluorescent protein in lentivirus-infected BMSCs. **c** Morphology of TERT-BMSCs. **d** Chemical structure of CRYYRITY. **e** Chemical structure of DSPE-PEG-CRYYRITY. **f** High-performance liquid chromatography (HPLC) analysis of CRYYRITY. **g** Mass spectrometry analysis of CRYYRITY. **h** Nuclear magnetic resonance spectrum of DSPE-PEG-CRYYRITY. Scale bar = 100 μm for all captured pictures.
